# Mycorrhizal Formation and Diversity of Endophytic Fungi in Hair Roots of *Vaccinium oldhamii* Miq. in Japan

**DOI:** 10.1264/jsme2.ME16011

**Published:** 2016-06-07

**Authors:** Takashi Baba, Dai Hirose, Nobumitsu Sasaki, Naoaki Watanabe, Nobuo Kobayashi, Yuji Kurashige, Fraidoon Karimi, Takuya Ban

**Affiliations:** 1 United Graduate School of Agricultural Science, Tokyo University of Agriculture and TechnologySaiwai-cho, Fuchu, Tokyo 3–5–8Japan; 2 School of Pharmacy, Nihon UniversityFunabashi, Chiba 274–8555Japan; 3 Gene Research Center, Tokyo University of Agriculture and TechnologySaiwai-cho, Fuchu, Tokyo 3–5–8Japan; 4 Faculty of Agriculture Field Science Center, Tokyo University of Agriculture and TechnologySaiwai-cho, Fuchu, Tokyo 3–5–8Japan; 5 Faculty of Life and Environmental Science, Shimane UniversityMatsue, Shimane 690–8504; 6 Niigata Prefectural Botanical GardenKanadzu, Akiha, Niigata 956–0845

**Keywords:** Clone library, dark septate endophyte, ericoid mycorrhizal fungi, hair root, *Vaccinium oldhamii* Miq

## Abstract

The root diameters as well as colonization and diversity of the root-associating fungi of *Vaccinium oldhamii* Miq. were investigated in order to obtain information on their mycorrhizal properties. The distal regions of roots had typical hair roots with diameters of less than 100 μm. Ericoid mycorrhizal fungi (ErMF) and dark septate endophytes (DSE) were frequently observed in the roots. Ascomycetes, particularly helotialean fungi, appeared to be dominant among the endophytic fungi of *V. oldhamii* roots. Furthermore, *Rhizoscyphus ericae* (Read) Zhuang & Korf and *Oidiodendron maius* Barron known as ErMF were detected more frequently than other fungal species.

*Vaccinium oldhamii* Miq. is a deciduous erect shrub that is widely distributed in Japan, Korea, and China. In Japan, it is found at the edges of broad-leaved forests in the lowlands to mountains in Hokkaido, Honshu, Shikoku, and Kyushu (24). *V. oldhamii* bears edible berries that are locally consumed fresh or processed. The fruits ripen after blueberry harvesting and have a larger amount of polyphenols and greater antioxidant activity than blueberries (16). *V. oldhamii* may be a useful breeding resource for developing new blueberry cultivars. However, its root characteristics have not yet been investigated.

Most ericaceous plants including *Vaccinium* species produce extremely fine absorptive roots referred to as hair roots that typically have diameters of less than 100 μm and lack root hairs (11). Hair roots are in symbiotic association with ericoid mycorrhizal fungi (ErMF) and form ericoid mycorrhizas (ErM). ErMF are mainly of ascomycetes and the representative species are *Rhizoscyphus ericae* (Read) Zhuang & Korf (syn. *Hymenoscyphus ericae*) forming “*R. ericae* aggregates” with closely related species in the order Helotiales and *Oidiodendron maius* Barron (9). ErMF mobilize recalcitrant organic nutrients, particularly nitrogen, and improve their host’s nutrient acquisition (9). ErMF are observed in wild and cultivated *Vaccinium* plants and inoculations with ErMF have been shown to improve the growth of cultivated blueberries (25). Therefore, the role of ErMF is important not only in the wild (11), but also in the horticultural production of *Vaccinium* plants, and a deeper understanding of the mycorrhizal properties of wild *Vaccinium* species is beneficial for the application of ErMF to the production of ericaceous crops such as blueberries.

Nineteen *Vaccinium* species have been identified in Japan (24); however, their mycorrhizal status is still unclear. In the present study, the root diameters as well as colonization and diversity of the root-associating fungi of *V. oldhamii* were investigated in order to improve our understanding on mycorrhizal formation.

## Materials and Methods

Plant samples were collected at the edges or interior of forests of *Quercus serrata* Thunb. or *Pinus densiflora* Sieb. et Zucc. in Shimane (July 2012), Kyoto (August 2012), Gunma (October 2012), Tokyo (November 2012) and Niigata (December 2012) in Japan (The locations of and detailed information on sampling sites are shown in Table S1). The soil pH of the sampling sites ranged between 4.1 and 4.6. Regardless of size or age, three healthy individual plants with lignified stems were randomly selected at the each sampling site and the roots of each individual were collected. Samples were transported to the laboratory, stored at 4°C, and processed within one week of sampling.

Roots were gently washed in tap water to remove adhering soil and organic debris. Root orders were determined based on the morphometric approach by Fitter (4) following Valenzuela-Estrada *et al.* (18). This approach considered 1^st^-order roots to be those terminating in a meristem and higher (n) order roots to be those extending from the junction of two same (n-1) order roots. Approximately 2 g of 1^st^- and 2^nd^-order roots were taken, washed three times with 10 mL of sterilized 0.05% aerosol OT (di-iso-octyl sodium sulfosuccinate) solution (w/v), and then rinsed three times with 10 mL of sterile distilled water. The washed roots were stored in 1.5 mL of 75% ethanol (v/v) until DNA extraction. The remaining roots were used for the observation of mycorrhizas.

Root samples for the observation of mycorrhiza were stained as previously described by Phillips and Hayman (10) with a slight modification based on Oba *et al.*, (7). Roots were soaked in 0.27% sodium-pyrophosphate solution (w/v) and cleaned in an ultrasonic bath for five min. The cleaned roots were cleared with 10% KOH (w/v) for two d, acidified with 1% HCl (v/v) for one d, and stained with 0.05% trypan blue lactic acid solution (w/v) for five d. These processes were completed at room temperature. The stained roots were observed under a light microscope (BX 50, Olympus, Tokyo, Japan) equipped with a digital camera (DP72, Olympus) at a magnification of 200 or 400×. Several types of structures were formed by ErMF and dark septate endophytes (DSE) in the root epidermal cells (Fig. S1). ErMF were identified as fine hyphal coils that developed inside epidermal or cortex cells, while the DSE association was recognized as intracellular microsclerotia and darkly pigmented thick septate hyphae (20). The presence or absence of ErMF or DSE structures in each randomly selected 500-μm-thick root section was confirmed. This operation was repeated 50 times on each order root and the percentage of colonized sections with ErMF or DSE was calculated by dividing the number of colonized root sections by 50 (*i.e.*, the total section number). We did not employ methods using line-intersection (*e.g.*, 5) to estimate fungal colonization levels because *V. oldhamii* roots were very fine and several different fungal structures were present close to each other (Fig. S1). Regarding samples used for mycorrhizal observations, the diameters of 1^st^- and 2^nd^-order roots were measured under a light microscope using image analysis software (DP2-BSW, Olympus). The mean diameter of each order root was calculated from 10 roots from each plant. The Student’s *t*-test was used for statistical comparisons of root diameters and colonization levels between root orders using Statcel3 (OMS publishing, Tokorozawa, Japan). Fungal colonization data were arcsine-transformed before statistical analyses.

The diversity of root-associating fungi was analyzed using the clone library method. An approximately 100-mg portion of hair roots was collected from each stored sample, frozen by liquid nitrogen, and homogenized with a pestle and mortar. Total genomic DNA was extracted using a DNA extraction kit (RBC Genomic DNA Extraction Kit Mini [Plant], RBC Bioscience, Taipei, Taiwan). The ITS regions of rDNA were amplified using a high-fidelity DNA polymerase (KOD FX Neo, Toyobo, Osaka, Japan) with the fungal-specific primers ITS1-F_KYO1 (5′-CTHGGTCATTTAGAGGAASTAA-3′; 15) and ITS4 (5′-TCCTCCGCTTATTGATATGC-3′; 23). A polymerase chain reaction (PCR) was performed under the following conditions: 10 min at 95°C; 35 cycles at 94°C for 20 s, at 58°C for 30 s, and at 72°C for 40 s, with a final extension at 72°C for 7 min. PCR products were cleaned using a DNA purification kit (QIAquick PCR Purification Kit, QIAGEN, Hilden, Germany) and then cloned using a DNA cloning kit (pGEM-T Easy Vector systems, Promega, Madison, WI, USA). Plasmid DNAs were isolated using a plasmid DNA extraction kit (HiYield™ Plasmid Mini Kit, RBC Bioscience) and at least 10 plasmids were obtained from each sample. Plasmids were sequenced by a DNA sequence service (Hokkaido System Science, Sapporo, Japan). In order to identify the closest matched fungal names, the sequences obtained were compared with those in GenBank through a BLAST search (http://blast.ncbi.nlm.nih.gov/). Contaminated plant DNA and sequences of inadequate quality were removed. Seventy-three fungal sequences were obtained, namely, five fungal sequences for each plant in each sampling site, except for one sample from Gunma with three sequences. All these sequences were clustered into operational taxonomic units (OTUs) with the BLASTClust program provided online by the Max Planck Institute (http://toolkit.tuebingen.mpg.de/blastclust#) based on 97% sequence similarity and 90% coverage criteria.

## Results and Discussion

The distal regions of *V. oldhamii* roots were typical hair roots with swollen epidermal cells and no root hairs (Fig. S1). The mean diameters of 1^st^- and 2^nd^-order roots ranged between 37.9 and 53.3 μm and between 59.1 and 74.3 μm, respectively (Table 1). Regarding the samples collected from Shimane and Niigata and the mean diameters of all sites, 2^nd^-order roots were significantly thicker than 1^st^-order roots. No significant differences were observed in root diameters between root orders in other sampling sites. The diameters of the 1^st^- and 2^nd^-order roots of *V. oldhamii* were consistent with those of the 1^st^- to 3^rd^-order roots of highbush blueberry ‘Bluecrop’, which ranged between 40 and 75 μm (18).

ErM was well developed in the hair roots of *V. oldhamii*. The colonization levels of ErMF ranged between 96.7 and 100% in the 1^st^-order roots and between 96 and 100% in the 2^nd^-order roots, while those of DSE ranged between 78 and 94.7% in the 1^st^ order roots and between 80 and 92.7% in the 2^nd^-order roots (Table 1). The mean colonization levels of ErMF in the overall sampling sites were 98.9% in the 1^st^-order roots and 98.3% in the 2^nd^-order roots, while those of DSE were 85.9% in the 1^st^-order roots and 87.2% in the 2nd-order roots. A comparison of ErMF and DSE colonization levels did not show any significant differences between 1^st^-and 2^nd^-order roots for each sampling site. In the present study, the “density of colonized cells” in 500-μm-thick root sections was not estimated. However, most root sections were normally colonized by ErMF (*e.g.* Fig. S1) and sparsely colonized sections were rarely observed. These results indicate the prevalence of ErMF and the DSE association with *V. oldhamii*. They also suggest that *V. oldhamii* nutrient uptake depends on the presence of ErMF and/or DSE.

Seventy-three fungal sequences from *V. oldhamii* roots were clustered into 35 OTUs (Table 2. The present study accession numbers were shown in Table S2). Of these, 65 sequences (89.0%) were assigned to Ascomycota and were clustered into 29 OTUs (82.9%) (Table 2, Fig. S2). At the order level, the most frequent ascomycetous taxon was Helotiales, which accounted for 52.1% of all sequences and 40% of all OTUs. Numerous ErMF species are included in the order Helotiales (8); Walker *et al.* (22) reported that 86.3 and 47.9% of the sequences obtained from three species of Arctic Ericaceae including *Vaccinium vitis-idaea* L. were assigned to Ascomycota and Helotiales, respectively.

The helotialean OTUs were found at all sampling sites (Table 2). OTU1 was the most frequent Helotiales species and was detected at all sites, except for Gunma. This sequence was highly identical to the Helotiales species isolated from the root epidermal cells of *Schizocodon soldanelloides* var. *magnus* (Diapensiaceae known to form ErM), which have hyphal coils and are considered to be ErMF (8) (Table S2). OTU4, found in Kyoto, Gunma, and Niigata, was the most closely related to the typical ErMF *R. ericae*. OTU8 was detected in Shimane, Kyoto, and Niigata. OTU9 was found in Shimane and Kyoto. OTU16 and OTU30 were found in Shimane and the closest matches of each OTU were *Meliniomyces* and *Acephala* species, which are known as ErMF and DSE, respectively (14, 21). OTU22, found in Kyoto, was the most closely related to *Cryptosporiopsis ericae* Sigler, reported to be ErMF (26). The other helotialean OTUs (OTU3, OTU7, OTU14, OTU17, OTU21, OTU34, and OTU35) were obtained from only one site, and their most closely related genus or species was not identified. Eleven sequences (15.1%) were categorized into four OTUs (11.4%) belonging to the genus *Oidiodendron*. Three OTUs (OTU2, OTU11, and OTU25) detected at all sites, except for Gunma, were closely related to the typical ErMF *O. maius*. OTU 27, found in Kyoto, was the most closely related to *Oidiodendron chlamydosporicum* Morrall, and was confirmed as an ErMF (3). Nine sequences (12.3%) were grouped into five OTUs (14.3%) belonging to the family Herpotrichiellaceae (Chaetothyriales). The Chaetothrialean OTUs (OTU6, OTU10, OTU13, OTU23, and OTU28) were detected at all sampling sites. OTU31 and OTU33, detected in Shimane, were the most closely related to *Cenococcum geophilum* Fr (Dothideomycetes, Mytilinidiales). This species are typical ectomycorrhizal fungi and known to associate with ericaceous roots (19). OTU15, found in Kyoto, was the most closely related to *Pseudocercosporella capsellae* (Ellis & Everh.) Deighton (Dothideomycetes, Capnodiales). OTU12 and OTU29, assigned to Dothideomycetes, were detected in Kyoto and Niigata. OTU20, found in Kyoto, was assigned to Sordariomycetes. In future research, these fungi, particularly helotialean endophytes, need to be isolated and their ability for mycorrhizal formation examined.

The percentage of Basidiomycetous sequences (9.6%) was markedly less than that of Ascomycota. No basidiomycetous ErMF, such as clade B sebacinalean fungi (12), were detected (Table 2, Fig. S2). Basidiomycetous OTUs were mainly found in Tokyo: OTU5 and OTU32 were assigned to Boletaceae and OTU19 to the genus *Russula*. OTU18, detected in Shimane, was the most closely related to *Trametes versicolor* (L.) Lloyd. OTU26, found in Gunma, was assigned to Tricholomataceae. Although basidiomycetes such as *Boletus* or *Russula* species have sometimes been found in ericoid roots, their frequency was low (1). Limited information is currently available on the relationship between basidiomycetes detected from *V. oldhamii* and other ericaceous plants. Therefore, it is not known whether these fungi are true fungal partners.

Only one sequence (OTU 24) was assigned to Glomeromycota (Table 2). This sequence, detected in Shimane, was closely related to *Rhizophagus diaphanus* (Morton and Walker) Schussler and was considered to be an arbuscular mycorrhizal fungus (AMF). Some structures such as vesicles and arbuscules formed by AMF were also observed in the samples collected from Shimane, Gunma, and Tokyo (Fig. S3). Although several studies have reported structures resembling those formed by AMF in the roots of ericaceous plants (*e.g.* 2, 6, 17), the relationship between ericaceous plants and AMF is not clearly understood. On the other hand, arbuscular mycorrhizal symbiosis is the most common mycorrhizal association and AMF are obligatory symbiotic fungi (13); therefore, AMF may be involved in the production of ericaceous crops. An inoculation test for AMF on *Vaccinium* species needs to be conducted.

In conclusion, *V. oldhamii* had typical hair roots with diameters of less than 100 μm. ErMF and DSE were frequently observed in the roots. Ascomycetes, particularly helotialean fungi, appeared to dominate among the endophytic fungi of *V. oldhamii* roots. Additionally, the sequences that were the most closely related to the representative ErMF species *R. ericae* and *O. maius* were more frequently detected than other fungal species.

## Supplementary Material



## Figures and Tables

**Table 1:** Root diameters and colonization levels of *Vaccinium oldhamii* Miq.

Site	Root diameter (μm)		Colonization level (%)			

			Ericoid mycorrhizal fungi		Dark septate endophytes	
		
	1st^a^	2nd^a^	1st	2nd	1st	2nd
Shimane	53.3 ±2.3^b^	74.3 ±5.0*c	100 ±0	100 ±0	94.7 ±2.4	89.3 ±0.7
Kyoto	48.9 ±5.3	70.7 ±5.8	100 ±0	98.7 ±1.3	88.7 ±4.8	80.0 ±3.1
Gunma	50.6 ±5.5	72.5 ±5.7	98.7 ±1.3	97.3 ±2.7	86.7 ±2.9	92.7 ±0.7
Tokyo	41.6 ±1.4	59.1 ±6.3	96.7 ±2.4	96.0 ±2.3	78.0 ±4.6	86.7 ±1.8
Niigata	37.9 ±1.6	60.3 ±4.4**	99.3 ±6.7	99.3 ±6.7	81.3 ±7.1	87.3 ±2.9
All sites^d^	46.5 ±2.9	67.4 ±3.2**	98.9 ±0.6	98.3 ±0.7	85.9 ±2.9	87.2 ±2.1

a Root order.

b Values are the mean ± SE (n=3) for all data except “All sites” data.

c * and ** reperesent significant differences (*P*<0.05 and <0.01, respectively) between root orders following the Student’s *t*-test. Fungal colonization data were arcsine-transformed before statistical analyses.

d Values are the mean ± SE of the five sampling sites (n=5).

**Table 2:** Clustering of 73 sequences as well as frequencies and BLAST results for 35 OTUs from the roots of *Vaccinium oldhamii* Miq.

OTU	Frequency (n)					BLAST results				
	
	Sn^a^	Kt	Gm	Tk	Ng	Closest match in GenBank (accession no.)	Order	Phylum^b^	Query coverage	Maximum identity
OTU1	4	1	0	3	5	Helotiales sp. (AB598101)	Helotiales	A	100%	99%
OTU2	1	2	0	1	3	*Oidiodendron maius (*KF359579)	incertae sedis	A	100%	99%
OTU3	0	0	5	0	0	Helotiales sp. (JQ272327)	Helotiales	A	100%	95%
OTU4	0	1	2	0	1	*Rhizoscyphus ericae (*JQ711893)	Helotiales	A	100%	97%
OTU5	0	0	0	3	0	Boletaceae sp. (HE814178)	Boletales	B	90%	100%
OTU6	1	0	0	0	2	Herpotrichiellaceae sp. (JQ272383)	Chaetothyriales	A	100%	97%
OTU7	0	0	0	3	0	Helotiales sp. (JQ272459)	Helotiales	A	99%	99%
OTU8	1	1	0	0	1	Helotiales sp. (AB847073)	Helotiales	A	90%	100%
OTU9	1	1	0	0	0	Helotiales sp. (JQ272327)	Helotiales	A	100%	96%
OTU10	0	1	0	0	1	Herpotrichiellaceae sp. (JQ272383)	Chaetothyriales	A	100%	99%
OTU11	0	1	0	1	0	*Oidiodendron maius (*HQ608115)	incertae sedis	A	100%	97%
OTU12	0	2	0	0	0	Dothideomycetes sp. (AB986427)		A	96%	83%
OTU13	0	0	1	1	0	Herpotrichiellaceae sp. (JQ272383)	Chaetothyriales	A	100%	88%
OTU14	0	1	0	0	0	Helotiales sp. (JQ272327)	Helotiales	A	100%	99%
OTU15	0	1	0	0	0	*Pseudocercosporella capsellae (*GU214662)	Capnodiales	A	100%	84%
OTU16	1	0	0	0	0	*Meliniomyces* sp. (EF093175)	Helotiales	A	92%	99%
OTU17	0	0	1	0	0	Helotiales sp. (KM113762)	Helotiales	A	98%	88%
OTU18	1	0	0	0	0	*Trametes versicolor (*JN164965)	Polyporales	B	99%	99%
OTU19	0	0	0	1	0	*Russula* sp. (AY750164)	Russulales	B	96%	95%
OTU20	0	0	1	0	0	Sordariomycetes sp. (GQ153124)		A	64%	87%
OTU21	0	0	1	0	0	Helotiales sp. (JQ272327)	Helotiales	A	98%	96%
OTU22	0	1	0	0	0	*Cryptosporiopsis ericae (*AY442322)	Helotiales	A	100%	99%
OTU23	0	1	0	0	0	Herpotrichiellaceae sp. (AB847033)	Chaetothyriales	A	96%	96%
OTU24	1	0	0	0	0	*Rhizophagus diaphanus (*AJ972462)	Glomerales	G	99%	86%
OTU25	0	0	0	1	0	*Oidiodendron maius (*KF359579)	incertae sedis	A	100%	93%
OTU26	0	0	1	0	0	Tricholomataceae sp. (KJ654632)	Agaricales	B	91%	91%
OTU27	0	1	0	0	0	*Oidiodendron chlamydosporicum (*NR_111032)	incertae sedis	A	90%	99%
OTU28	0	0	0	0	1	Herpotrichiellaceae sp. (KF359595)	Chaetothyriales	A	100%	96%
OTU29	0	0	0	0	1	Dothideomycetes sp. (AB986427)		A	95%	84%
OTU30	1	0	0	0	0	*Acephala* sp. (KC480052)	Helotiales	A	97%	97%
OTU31	1	0	0	0	0	*Cenococcum geophilum (*JQ711896)	Mytilinidiales	A	77%	94%
OTU32	0	0	0	1	0	Boletaceae sp. (HE814178)	Boletales	B	90%	100%
OTU33	1	0	0	0	0	*Cenococcum geophilum (*JQ711896)	Mytilinidiales	A	100%	99%
OTU34	1	0	0	0	0	Hyaloscyphaceae sp. (JQ272392)	Helotiales	A	93%	97%
OTU35	0	0	1	0	0	Helotiales sp. (JQ272334)	Helotiales	A	100%	90%

a Sn: Shimane, Kt: Kyoto, Gm: Gunma, Tk: Tokyo, Ng: Niigata.

b A: Ascomycota, B: Basidiomycota, G: Glomeromycota.
