# Supplementary material for: Mycorrhizal Formation and Diversity of Endophytic Fungi in Hair Roots of Vaccinium oldhamii Miq. in Japan
Source: Microbes Environ. 2016 Jun 7;31(2):186–9. doi: 10.1264/jsme2.ME16011 (PMC4912157; doi:10.1264/jsme2.ME16011)
Supplement: Supplementary file 1 [file 31_186_s1.pdf]

Table S1 Geographical location and description of sampling sites

Site	Latitude, Longitude	Altitude (m)[a]	Site types[b]	Dominant tree species[c]	pH
Shimane	35°30 N, 133°06 E	40	E	Q	4.6 ±0.0
Kyoto	35°35 N, 135°01 E	130	E	P	4.5 ±0.1
Gunma	36°33 N, 139°24 E	920	E	Q	4.1 ±0.0
Tokyo	35°38 N, 139°22 E	170	I	Q	4.1 ±0.0
Niigata	37°45 N, 139°06 E	40	E	Q	4.5 ±0.0

[a] Approximately altitudes of sampling sites are shown by the 10m.

[b] E, edge of forest; I, interior of forest.

[c] Q, *Quercus serrata* Thunb.; P, *Pinus densiflora* Sieb. et Zucc.

Table S2 Accession numbers and isolation sources, reference and endophytic status of Closest match in GenBank for 35 OTUs from the roots of *Vaccinium oldhamii* Miq.

OTU	Representative accession no. in GenBank	Closest match in GenBank (accession no.)	Isolation source (Additional reference)	Reference	Endophytic status[a] (Additional reference)
OTU1	KU550986	Helotiales sp. (AB598101)	*Schizocodon soldanelloides* var. *magnus* root cell	(16)	ErM
OTU2	KU550981	*Oidiodendron maius* (KF359579)	Root	(1)	ErM (17)
OTU3	KU550972	Helotiales sp. (JQ272327)	Root	(2)	RE?
OTU4	KU550968	*Rhizoscyphus ericae* (JQ711893)	Ectomycorrhizal root tip	(12)	ErM (17)
OTU5	KU551037	Boletaceae sp. (HE814178)	Ectomycorrhizal root tip	(8)	EcM
OTU6	KU551007	Herpotrichiellaceae sp. (JQ272383)	Root	(2)	RE?
OTU7	KU551028	Helotiales sp. (JQ272459)	Root	(2)	RE?
OTU8	KU550989	Helotiales sp. (AB847073)	*Enkianthus campanulatus* root	(15)	RE?
OTU9	KU550980	Helotiales sp. (JQ272327)	Root	(2)	RE?
OTU10	KU550982	Herpotrichiellaceae sp. (JQ272383)	Root	(2)	RE?
OTU11	KU550994	*Oidiodendron maius* (HQ608115)	*Trachymyrmex septentrionalis* nest	(18)	ErM (17)
OTU12	KU550991	Dothideomycetes sp. (AB986427)	Black sclerotium	(14)	U
OTU13	KU550969	Herpotrichiellaceae sp. (JQ272383)	Root	(2)	RE?
OTU14	KU550985	Helotiales sp. (JQ272327)	Root	(2)	RE?
OTU15	KU550984	*Pseudocercosporella capsellae* (GU214662)	*Brassica* sp.	(5)	E (4)
OTU16	KU551013	*Meliniomyces* sp. (EF093175)	*Picea abies* ectomycorrhizal root tip	(22)	ErM
OTU17	KU550967	Helotiales sp. (KM113762)	Depleted uranium in soil	Fomina and Gadd (unpublished)	U
OTU18	KU551012	*Trametes versicolor* (JN164965)	*Nothofagus antarcticae* (24)	(13)	U
OTU19	KU551036	*Russula* sp. (AB972834)	*Pinus densiflora* ectomycorrhizal root tip	(7)	EcM
OTU20	KU550970	Sordariomycetes sp. (GQ153124)	*Juniperus deppeana* photosynthetic tissue	(11)	E?
OTU21	KU550978	Helotiales sp. (JQ272327)	Root	(2)	RE?
OTU22	KU550987	*Cryptosporiopsis ericae* (AY442322)	*Gaultheria shallon* ericoid mycorrhizal root	(19)	ErM (25), DSE (23)
OTU23	KU550988	Herpotrichiellaceae sp. (AB847033)	*Enkianthus campanulatus* root	(15)	RE?
OTU24	KU551019	*Rhizophagus diaphanus* (AJ972462)	Spore	(3)	AM[b]
OTU25	KU551026	*Oidiodendron maius* (KF359579)	Root	(1)	ErM (17)
OTU26	KU550977	Tricholomataceae sp. (KJ654632)	*Acacia mangium* root	(9)	RE?
OTU27	KU550993	*Oidiodendron chlamydosporicum* (NR_111032)	Boreal forest soil	(10)	ErM (6)
OTU28	KU551006	Herpotrichiellaceae sp. (KF359595)	Root	(1)	RE?
OTU29	KU551009	Dothideomycetes sp. (AB986427)	Black sclerotium	(14)	U
OTU30	KU551020	*Acephala* sp. (KC480052)	*Picea abies* root	(20)	DSE
OTU31	KU551021	*Cenococcum geophilum* (JQ711896)	Ectomycorrhizal root tip	(12)	EcM, RE (21)
OTU32	KU551035	Boletaceae sp. (HE814178)	Ectomycorrhizal root tip	(8)	EcM
OTU33	KU551016	*Cenococcum geophilum* (JQ711896)	Ectomycorrhizal root tip	(12)	EcM, RE (21)
OTU34	KU551010	Hyaloscyphaceae sp. (JQ272392)	Root	(2)	RE?
OTU35	KU550979	Helotiales sp. (JQ272334)	Root	(2)	RE?

[a] AM: Arbuscular mycorrhizal, DSE: Dark septate endophytic, E: Endophytic, EcM: Ectomycorrhizal, ErM: Ericoid mycorrhizal, RE: Root endophytic, U: Unknown. When the endophytic statuses are not clear in the BLAST references, question marks are added. Additional references are shown as necessary.

[b] International Culture Collection of Arbuscular and Vesicular-Arbuscular Mycorrhizal Fungi. West Virginia University. http://www.invam.caf.wvu.edu/. See *Rhizophagus diaphanus*.

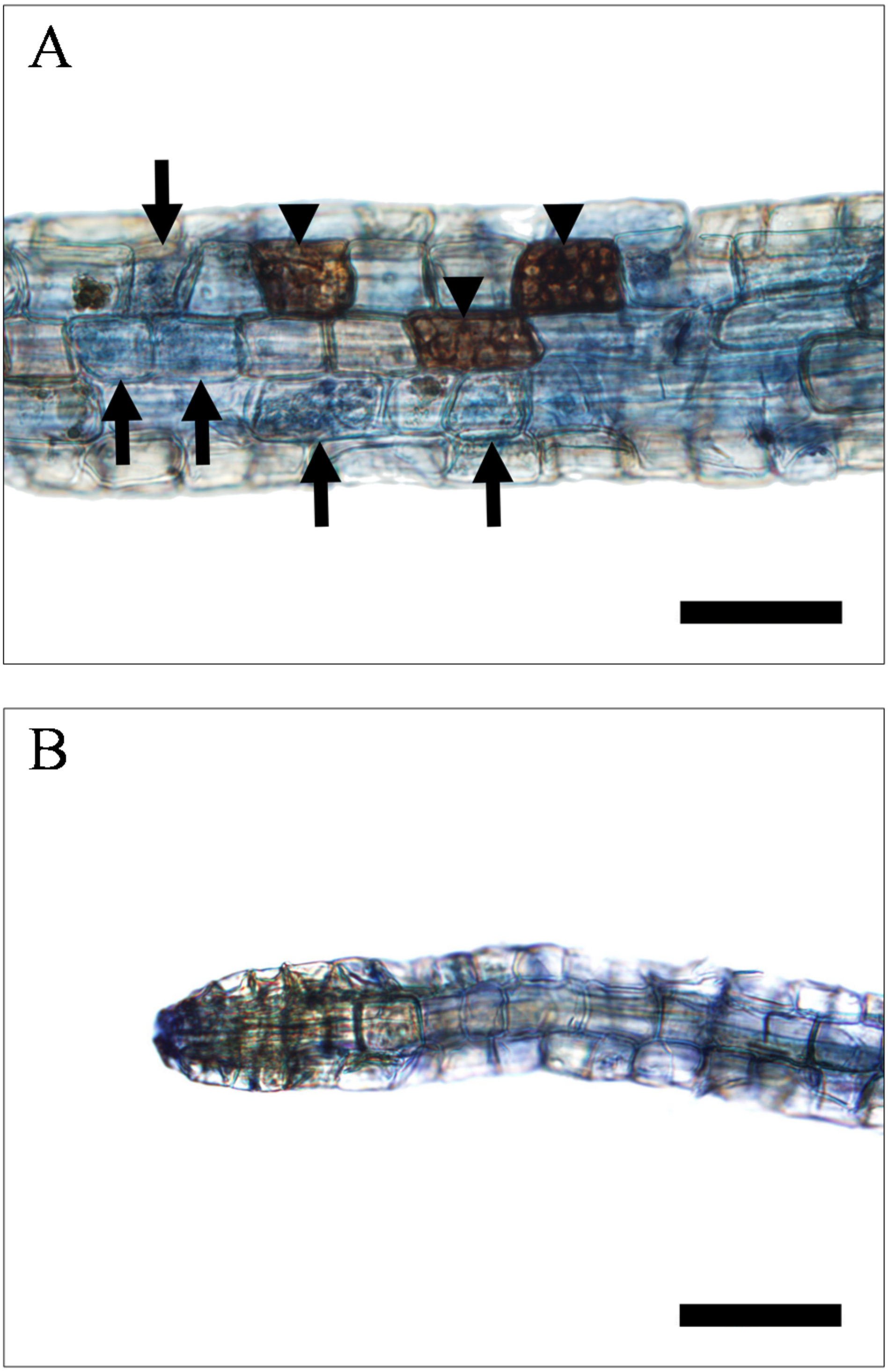

Fig. S1. Morphology of hair roots of *Vaccinium oldhamii* Miq. and internal fungal structures. **(A)** Fine hyphal coils of ericoid mycorrhizal fungi (arrows) and pigmented microsclerotia of dark septate endophytes (arrow heads) in a hair root. Bar = 50 µm. (**B)** A distal region of *V. oldhamii* root. Bar = 50 µm.

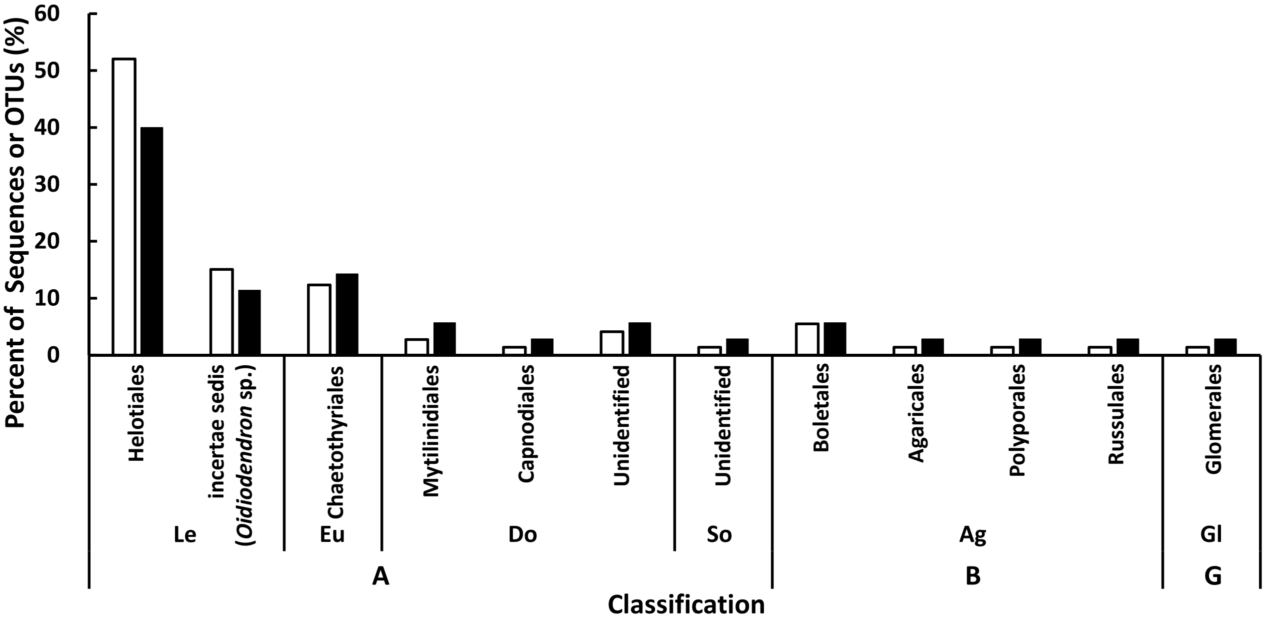

Fig. S2. Percent of sequences (open bars) and OTUs (closed bars) belonging to each fungal order from *Vaccinium oldhamii* Miq. roots.

Abbreviation: Leotiomycetes; Le, Eurotiomycetes; Eu, Dothideomycetes; Do, Sordariomycetes; So, Agaricomycetes; Ag, Glomeromycetes; Gl, Ascomycota; A, Basidiomycota; B, Glomeromycota; G.

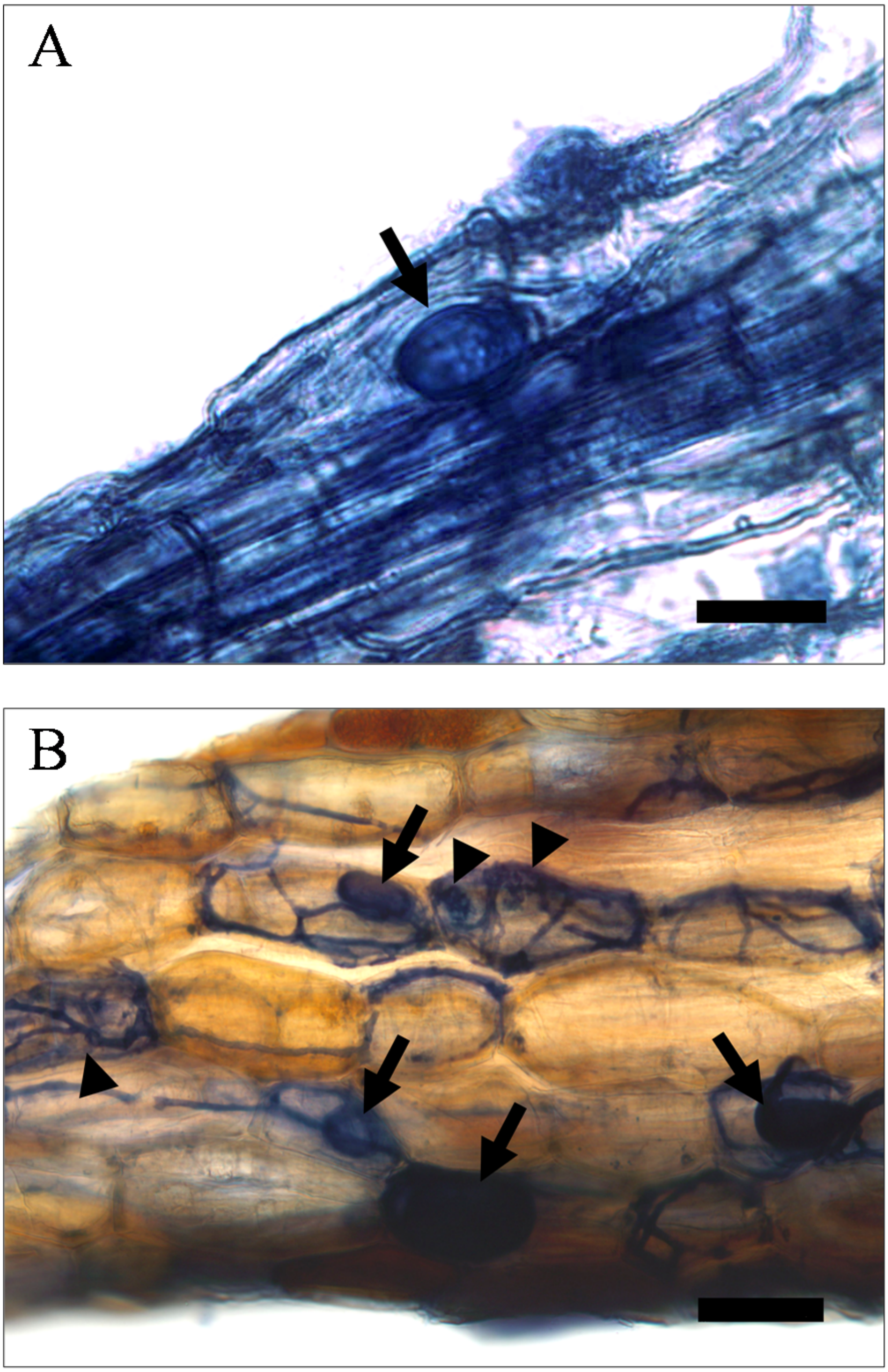

Fig. S3. Structures like arbuscular mycorrhizal fungi observed in roots of *Vaccinium oldhamii* Miq. **(A)** A structure (arrow) like vesicle in a broken hair root. Bar = 20 µm. **(B)** Structures like vesicles (arrows) and arbuscules (arrow heads) in a coarse root. Bar = 50 µm.

Supplementary references

1. Baired, R., C. E. Stokes, A. Wood-Jones, C. Watson, M. Alexander, G. Taylor, K. Johnson, P. Threadgill and S. Diehl. 2014. A molecular clone and culture inventory of the root fungal community associated with eastern hemlock in Great Smoky Mountain national park. Southeastern Naturalist 13: 219-237.
2. Baird, R., Wood-Jones, A., Varco, J., Watson, C., Starrett, W., Taylor, G., Johnson, K. 2014. Rhododendron decline in the Great Smoky Mountain and surrounding areas: intensive site study of biotic and abiotic parameters associated with the decline. Southeastern Naturalist 13: 1-25.
3. BŁaszkowski, J., C. Renker and F. Buscot. 2006. *Glomus drummondii* and *G. walkeri*, two new species of arbuscular mycorrhizal fungi (Glomeromycota). Mycol. Res. 110: 555-566.
4. Boerema, G. H, and A. A. Verhoeven. 1980. Check-list for scientific names of common parasitic fungi. Series 2d: Fungi on field crops: vegetables and cruciferous crops. Neth. J. Plant Pathol. 86: 199-228.
5. Crous, P. W., C.L. Schoch, K.D. Hyde, A.R. Wood, C. Gueidan, G.S. de Hoog and J.Z. Groenewald. Phylogenetic lineages in the Capnodiales. Stud. Mycol. 64: 17-47.
6. Daipé, Y. 1991. Statut endomycorrhizien du genre Oidiodendron. Can. J. Bot. 69 1712-1714.
7. Fukasawa, Y. 2015. Basidiomycetous ectomycorrhizal fungal communities of current-year Pinus densiflora seedlings that regenerated on decayed logs and on the forest floor soil. Journal of Integrated Field Science 12: 19-30.
8. Gao, C., N.-N. Shi, Y.-X. Liu *et al.* 2013. Host plant genus-level diversity is the best predictor of ectomycorrhizal fungal diversity in a Chinese subtropical forest. Mol. Ecol. 22: 3403-3414.
9. Glen, M., V. Yuskianti, D. Puspitasari, A. Francis, L. Agustini, A. Rimbawanto, H. Indrayadi, A. Gafur and C. L. Mohammed. 2014. Identification of basidiomycete fungi in Indonesian

hardwood plantations by DNA barcoding. Forest Pathology 44: 496-508.

10. Hambleton, S., K. N. Egger, R and S. Currah. The genus *Oidiodendron*: species delimitation and phylogenetic relationships based on nuclear ribosomal DNA analysis. Mycologia 90: 854-869.
11. Hoffman, M. T. and A. E. Arnold. 2010. Diverse bacteria inhabit living hyphae of phylogenetically diverse fungal endophytes. Appl. Environ. Microbiol. 46: 4063-4075.
12. Jones, M., D., L. A. Phillips, R. Treu, V. Ward and S. M. Berch. 2012. Functional responses of ectomycorrhizal fungal communities to long-term fertilization of lodgepole pine (*Pinus contorta* Dougl. ex Loud. var. *latifolia* Engelm.) stands in central British Columbia. Applied Soil Ecology 60: 29-40.
13. Justo, A. and D. S. Hibbett. 2011. Phylogenetic classification of *Trametes* (Basidiomycota, Polyporales) based on a five-marker dataset. Taxon.60: 1567-1583
14. Obase, K. G. W. Douhan, Y. Matsuda and M. E. Smith. 2014. Culturable fungal assemblages growing within *Cenococcum* sclerotia in forest soils. FEMS Microbiol. Ecol. 90: 708-717.
15. Obase, K. and Y. Matsuda. 2014. Culturable fungal endophytes in roots of *Enkianthus campanulatus* (Ericaceae). Mycorrhiza. 24: 635-644.
16. Okuda, A., M. Yamato, and K. Iwase. 2011. The mycorrhiza of *Schizocodon soldanelloides* var. *magnus* (Diapensiaceae) is regarded as ericoid mycorrhiza from its structure and fungal identities. Mycoscience. 52: 425-430.
17. Perotto, S., E. Martino, S. Abbá, and M. Vallino, 2012. 14 Genetic diversity and functional aspect of ericoid mycorrhizal fungi. p. 255-285, *In*: B. Hock (ed.). The Mycota Volume9 Fungal Associations. A comprehensive treatise on fungi as experimental systems for basic and applied research. Springer, Berlin, Heidelberg.
18. Rodrigues, A., U. G. Mueller, H. D. Ishak, M. Bacci Jr and F. C. Pagnocca. 2011. Ecology of microfungal communities in gardens of fungus-growing ants (*Hymenoptera: Formicidae*): a

year-long survey of three species of attine ants in Central Texas. FEMS Microbiol. Ecol. 78: 244-255

19. Sigler, L. T. Allan, S. R. Lim, S. Berch and M. Berbee. 2005. Two new *Cryptosporiopsis* species from roots of ericaceous hosts in western North America. Stud. Mycol. 53: 53-62.
20. Terhonen, E., S. K. H. Sun and F. O. Asiegbu. 2014. Endophytic fungi of Norway spruce roots in boreal pristine mire, drained peatland and mineral soil and their inhibitory effect on Heterobasidion parviporum in vitro. Fungal Ecology 9: 17-26.
21. Vohník,M., M. Fendrych, J. Albrechtová and M. Vosátka. 2007. Intracellular colonization of *Rhododendron* and *Vaccinium* roots by *Cenononnum geophilum*, *Geomyces pannorum* and *Meliniomyces variabilis*. Folia Microbiol. 52: 407-414.
22. Vohník,M., L. Mrnka, T. Lukešova, M. C. Bruzone, P. Kohout and J. Fehrer. The cultivable endophytic community of Norway spruce ectomycorrhizas from microhabitats lacking ericaceous hosts is dominated by ericoid mycorrhizal *Meliniomyces variabilis.* Fungal Ecology 6: 281-292.
23. Wang, W., A. Tsuneda, C. F. Gibas and R. S. Currah. 2007. Cryptosporiopsis species isolated from the roots of aspen in central Alberta: identification, morphology, and interactions with the host, in vitro. Can. J. Bot. 85: 1214-1226.
24. Wright, J. E. and J. R. Deschamps. 1972. Basidiomycetes xilófagos de los bosques andinopatagónicos. Rev. Invest. Agropecu., Ser. 5 9: 111-195.
25. Zhang, C., L. Yin, and S. Dai. 2009. Diversity of root-associated fungal endophytes in *Rhododendron fortunei* in subtropical forests of China. Mycorrhiza. 19: 417-423.
